# Capturing Land for Elephant Corridors in South India through the Conservation-Agrarian Squeeze

**DOI:** 10.1007/s00267-025-02192-4

**Published:** 2025-06-04

**Authors:** Ananda Siddhartha

**Affiliations:** 1 Sociology of Development and Change, Wageningen University & Research, Wageningen, the Netherlands; 2 Ashoka Trust for Research in Ecology and the Environment, Bangalore, Karnataka India

**Keywords:** Grab, Dispossession, Agrarian change, Conservation-agrarian squeeze, Elephant corridor, India

## Abstract

Contemporary conservation visions stress the need to expand land for biodiversity protection globally, despite many critiques saying that this often leads to human dispossession from land and resources. Recent global goals focus on ‘30 × 30’: extending conservation spaces to 30% of the globe by 2030, rendering the question of how to deal with the many people that inevitably live on these lands acute. While not solely reliant on protected areas, this approach incorporates various land types to meet this target, potentially including restrictions on its use. In India, acquiring land for elephant corridors is one example of extending conservation spaces into surrounding agrarian landscapes. This research investigates such a case in South India where farmers whose lands are identified for acquisition already struggle with challenges in the agrarian landscape, including neglect of agriculture by the state, rising financial debt, uncertain and changing weather patterns. Land use restrictions around protected areas, along with lack of compensation for wildlife-induced crop losses, have increased livelihood pressures, forcing farmers to diversify their income sources. Building on the conservation and agrarian literature, this article posits the concept of the ‘Conservation-Agrarian Squeeze’ (CAS) to make sense of the dual forces acting on farmers. This concept describes cases where land enclosure for conservation beyond PAs is facilitated by distress in agrarian landscapes. It also engages with and builds on existing terms such as the grab, induced volition, rendering surplus, and expulsion.

## Introduction

There is a global push to extend conservation frontiers. Drawing from E.O. Wilson’s (2016) ‘half earth’ idea and broader ‘nature needs half’ networks that call for half the earth to be designated into Protected Areas (PAs), the recent CBD COP 15 agreed to conserve 30 percent of land and seas by 2030, commonly referred to as ‘30 × 30’. In India, the rapid growth of PAs from 65 in 1970 to over 1100 in 2025^1^ highlights the continued focus on area-based conservation. Despite this increase, PAs cover 5.28% of the Indian land area and increasing this coverage is extremely challenging (Krishnamurthy 2023). This makes India’s aim to achieve the 30 × 30 target near impossible, except through so-called ‘Other Effective Area-Based Conservation Measures’ (OECMs) or areas other than PAs, that provide long-term in-situ biodiversity conservation. A document on identifying OECMs in India states that “OECMs present an opportunity to expand India’s network of conserved areas, operating cooperatively with protected areas” (MoEFCC, NBA and UNDP 2022 p. 5). It also emphasizes that they should effectively safeguard “areas of importance for ecological connectivity or that are important to complete a conservation network within a landscape or seascape” (ibid. p. 15) Although not yet implemented, increasing pressure to incorporate various land types around formal PAs is likely in the coming years, particularly if the land is deemed crucial for elephants or tigers.

Beyond meeting international targets, various approaches have been proposed in India to expand conservation beyond PA boundaries, often into surrounding agrarian landscapes. For example, the ‘Tiger Habitat Expansion Model’ suggested that farmers near PAs leave land uncultivated, gaining more from tourism than farming while benefiting themselves, tourists, and tiger (*Panthera tigris*) habitats (Karanth and Karanth 2012). In the state of Karnataka, where this study is based, the forest department proposed private conservancies in 2018, allowing private landholdings of at least 100 acres adjoining a PA to be set aside for conservation and wildlife connectivity^2^. Though unimplemented, these proposals underscore efforts to expand conservation into agrarian landscapes through eco-tourism, effectively “rendering land touristifiable” (Pandya et al. 2023). Prior to these proposals, regulations were introduced governing land around PAs through two mechanisms: Ecological Sensitive Zones (ESZs) and buffer areas for tiger reserves. Introduced in the 1990s, ESZs regulate activities within a specified distance of a PA boundary. However, restrictions introduced can have negative effects on agrarian landscapes, as I will discuss in the following sections.

Acquiring agricultural land for elephant corridors is another approach to expanding conservation areas and improving connectivity between PAs. Said to be “an essential conservation accessory” (Worboys et al. 2016 p. 6), corridors are ‘secured’^3^ through this method (Menon 2005; Menon 2017). The Asian elephant (*Elephas maximus*) is used as a flagship species to mobilise public and financial support for corridors (Barua et al. 2010; Jepson and Barua 2015) and are supported through celebrity advocacy (Abidin et al. 2020), art and charity events (Barua 2014), fundraising appeals (IFAW n.d.) and grants from international conservation organisations focused on land acquisition (IUCN-NL 2020).

These acquisitions do not occur in isolation, but within an agrarian context transformed by India’s economic liberalization since the early 1990s. Unfavourable policies, coupled with climate uncertainties, have slowed the agricultural economy, deepening livelihood and food insecurity for many who rely on farming (Posani 2009; Vasavi 2009; Kamra 2022). Consequently, rural livelihoods have become increasingly reliant on fragmented and insecure sources (Akram-Lodhi and Kay 2010), leaving many to abandon agriculture in pursuit of “post-agrarian futures” (Hall et al. 2011). State neglect of this sector has also led to the financialization of agrarian land, compelling farmers to sell, resulting in “dispossession by neglect” (Vijayabaskar and Menon 2018). While scholars have highlighted the influence and role of conservation in exacerbating agrarian differentiation and change through (eco)tourism, infrastructural development and payment for ecosystem services (Dressler 2011; Dressler et al. 2013; Pandya et al. 2023), I develop the idea of a ‘Conservation-Agrarian Squeeze’ (CAS) to explore how conservation interventions and restrictions work in conjunction with dynamics in agrarian landscapes to compel farmers to sell their land for conservation.

Using the acquisition of land for three identified elephant corridors^4^ connecting the Biligiri Rangaswamy Temple (BRT) Tiger Reserve in Karnataka, South India, to adjoining PAs as an empirical case, this article demonstrates how the CAS is manifested. What follows is a discussion on land and green grabs, regimes of dispossession, restrictions and the role of neglect in dispossession. After defining the CAS and building on concepts similar to the squeeze, I empirically identify five factors that contribute to and enable the acquisition of land for elephant corridors. I conclude by revisiting how the increasing interest to expand conservation spaces could result in many more examples of the CAS.

## Conceptual Framework

Globally and in India, scholarly research on land acquisition for various purposes and its associated dispossession has been increasing. Since 2006, there has been growing academic focus on the ‘global land grab’ (Borras et al. 2012; Hall 2013; Wolford et al. 2013) and its subset, known as green grabbing (Fairhead et al. 2012). Scholars have highlighted how the latter has contributed to the dispossession of people from their land and resources, undermined local sovereignty, and leveraged ‘green’ credentials for otherwise declining forms of capital accumulation (Benjaminsen and Bryceson 2012; Corson and MacDonald 2012).

In India, the 1990s saw a shift from state-managed capitalism, where land was acquired for public-sector projects, to neoliberalism, where land was taken for private corporations and Special Economic Zones (Levien 2018). Using ‘development’ as a justification for dispossession, Levien refers to “regimes of dispossession”, where the political apparatus was used to coercively acquire land for specific economic purposes and class interests (2018 p. 17). Kabra and Das (2022) utilise Levien’s regimes of dispossession to critically analyse how the continued push for inviolate areas for the tiger has been used to dispossess for conservation. While both Levien, and Kabra and Das highlight the role of the state in dispossession, NGOs are also essential actors within these regimes (Bathija and Sylvander 2023).

In India, the acquisition of land for conservation by NGOs, which is not widely practiced, is aided by the imposition of conservation restrictions on rural livelihoods by the state, thus increasing dependence on wage labour (Brockington and Duffy 2010). Restricting access, zonation exercises around PAs regulating land use, and banning customary grazing and resource collection increase the precarity of those living near PAs (Cernea 2005; DeFries et al. 2007; Hall et al. 2011). Over time, increasing restrictions can make land ‘grabbable’ and people ‘relocatable’ (Weldemichel 2022), leading to conservation dispossessions in agrarian landscapes.

Scholars have also highlighted that neglect can facilitate dispossession (Lima and Kmoch 2021), as seen in state underinvestment in agriculture and the financialization of land (Vijayabaskar and Menon 2018). Through the twin processes of neglecting agriculture and liberalising land markets in these landscapes, farmers are effectively coerced to sell their land due to the state favoring non-agricultural modes of accumulation, leading to what Vijayabaskar and Menon term ‘dispossession by neglect’. Policy shifts in agriculture and agrarian dynamics can blur the line between coercive and voluntary land sales, especially when conservation actors acquire land, and land use shifts from agriculture to state-owned conservation areas.

It is here that I introduce the Conservation-Agrarian Squeeze, where I conceptualise it as the convergence of factors and forces arising out of conservation and the agrarian that impinge upon and alter people’s livelihoods and support systems due to immense pressures. This idea relates to and builds on four existing and related terms – the grab, induced volition, rendering surplus and expulsion – which I briefly engage with below.

The word grab has been associated with terms such as land and green grabbing since it was first used by GRAIN, a civil society organisation, following foreign land acquisitions in Latin America in 2008 (Goetz 2021). A land grab can be defined as the unfair appropriation of land that “occur under conditions of highly asymmetric power relations, access to information, and distribution of benefits and costs” (Margulis et al. 2013, p. 16). Similarly, a green grab “does not always involve the wholesale alienation of land from existing claimants,” but involves “the restructuring of rules and authority over the access, use and management of resources, in related labour relations, and in human-ecological relationships, that may have profoundly alienating effects” (Fairhead et al. 2012 p. 239). As I will show, the squeeze exhibits features of both land and green grabs. However, land acquisition is a process facilitated by a combination of factors and forces within conservation and agrarian landscapes that enable the process.

Discursively reframing dispossession as voluntary does not account for the conditions created to bring about the acceptance to relocate, i.e. inducing volition (Milgroom and Spierenburg 2008; Bathija and Sylvander 2023). According to Milgroom and Spierenburg, induced volition occurs when restrictions on livelihood strategies imposed by park regulations coupled with increased wildlife presence, pressures some communities into ‘accepting’ resettlement. In India, such volition has and continues to be assembled by the hegemony and authority of the state to create inviolate areas for tiger conservation (Kabra and Das 2022). In this case, non-state conservation actors induce volition to acquire land, aided by state-imposed restrictions on livelihood strategies within and around BRT, such as the establishment of an ESZ, buffer areas, and limitations on access. Hence, the squeeze, as I conceptualise it includes inducement to sell land but is aided by a number of other factors. While Milgroom and Spierenburg do briefly touch upon the effect of wildlife on agriculture, other dynamics in agrarian landscapes that also contribute to dispossession and the squeeze are not factored in.

The third concept that has similarities with the squeeze is that of rendering populations as surplus (Li 2010). Dispossession in Southeast Asia has been the result of largescale enclosures for agricultural expansion, conservation, extractive industries, and the gradual dispossession of farmers through mounting debt, referred to as ‘intimate exclusions’ (Howson 2017). Here, rendering populations as surplus is framed in specific reference to capital accumulation and its requirements in specific locations. What differentiates the squeeze is the multiplicity of factors, which I will elaborate on below, in conservation and agrarian landscapes, including debt as Li points out, that enables dispossession. Furthermore, largescale agricultural expansion is not a feature in this landscape. Instead, there exist multiple overlapping forces that squeeze small and marginal farmers.

Finally, the term expulsion mirrors Levien’s (2018) ‘regimes of dispossession’. As Sassen notes (2014 p. 1), expulsion “takes us beyond the more familiar idea of growing inequality as a way of capturing the pathologies of today’s global capitalism” and “brings to the fore the fact that forms of knowledge and intelligence we respect and admire are often at the origin of long transaction chains that can end in simple expulsions.” Levien similarly highlights how different types of knowledge intersect with political and economic forces to dispossess peasants from their land. Sassen on the other hand highlights how advanced political economies, with complex financial instruments, land acquisition, and resource extraction, can result in elementary brutalities and expulsion. While this complexity cannot be pinned to an obvious centre or oppressor, there are sites where it all comes together. It is here that the squeeze builds on expulsion. In addition to forms of knowledge, complexities and the combination of economic forces coming together leading to expulsion, a conservation logic is also present. The extension of the conservation paradigm governs agrarian landscapes indirectly through the imposition of rules and restrictions, but also directly through the capture of agricultural land for elephant corridors.

In summary, I have highlighted how coercion often plays a key role in setting aside land for development, conservation, or other purposes, particularly in relation to the concepts of grab and induced volition. The concept of rendering surplus, though closely linked to the idea of the squeeze, primarily emphasize large-scale agricultural expansion and capital accumulation as drivers of dispossession. In the case of expulsion, what is missing from the analysis of knowledge systems and economic forces is the role of the conservation logic in expanding protected spaces into surrounding landscapes.

## Methods

To understand how agricultural land is acquired for elephant corridors and the broader context in which this occurs, empirical data were collected in the buffer and ESZ around BRT from December 2021 to July 2022. Qualitative data were collected through a combination of 50 semi-structured and structured interviews with small and marginal farmers, including those whose land was acquired for a corridor, agricultural labourers, representatives of the forest department, department of agriculture, revenue department and *panchayat*^5^ members. Observations also contributed to the data collected. Secondary data were collected through an analysis of reports and documents from the forest department and conservation organizations.

The interviews covered a broad range of topics. On conservation, information was gathered on prior and current interactions with BRT and the forest department, including how restrictions over time have impacted livelihoods and support systems. Additionally, views on elephant corridors and land acquisition for them, as well as changes in the landscape following land acquisitions, were also explored. On agrarian dynamics, historical changes in cropping patters, support by the state, issues facing farmers, including those by the forest department and wildlife, market dynamics etc were discussed. Finally, interviewees were also asked about their perspectives on the future of conservation and agriculture in the landscape. Verbal informed consent was sought from all interviewees prior to interactions and their anonymity was guaranteed.

This study faced two methodological limitations: first, achieving a gender balance among respondents was not possible due to my positionality as an urban male (albeit from Karnataka), and the challenges of engaging openly with women because of existing gender dynamics. Second was the inability to locate and speak to families whose land was acquired for the Edeyarahalli – Doddasampige corridor in 2003 since data for this research were collected almost two decades later.

Before presenting the empirical data, I will briefly outline the categories of PAs in India, explain how elephant corridors fit in, and describe BRT to provide context for Indian conservation efforts and the study area.

## Conservation in India

### Protected Areas and Elephant Corridors

India has four categories of terrestrial PAs under the Wildlife Protection Act (WLPA), 1972 – national parks, wildlife sanctuaries, conservation reserves and community reserves, which prioritise area-based conservation. Tiger reserves were added in 1973 as an additional designation to national parks and wildlife sanctuaries in response to a task force report that urged the government to create ‘safe havens’ for the tiger. These reserves are conferred the highest status of protection and are required to be inviolate. Consequently, since the early 1970s, forest dwellers have been relocated to the fringes of PAs by the forest department. Additionally, access to forest resources has been restricted not only for forest dwellers but also for those in surrounding landscapes who depended on them.

Alongside the growing number of PAs in the country, there has been growing attention to connectivity, in line with global efforts to prevent them from becoming isolated ‘islands’ amid the threat of fragmentation. In India, this effort began in the 1980s with the mapping of elephant corridors in various locations across the country (Sukumar 1985; Johnsingh et al. 1990; Johnsingh and Williams 1999) shaping the early discourse on connectivity and corridor integrity. A few decades later, a report identifying 88 corridors across the sub-continent was published (Menon 2005). Since then, their significance has been highlighted by the national level Report of the Elephant Task Force (Rangarajan et al. 2010), and the number of corridors has risen to 101 in 2017 (Menon 2017) and 150 in 2023 (Project Elephant Division, MoEF&CC, Government of India 2023). These corridors have been ‘secured’ through various means, including land acquisition, which I will elaborate on. However, these acquisitions have not occurred in isolation; they have unfolded within an agrarian context shaped by multiple dynamics that, as I argue, have influenced these processes. I will explore these aspects in the following sections.

### Biligiri Rangaswamy Temple Tiger Reserve

**Fig. 1:**
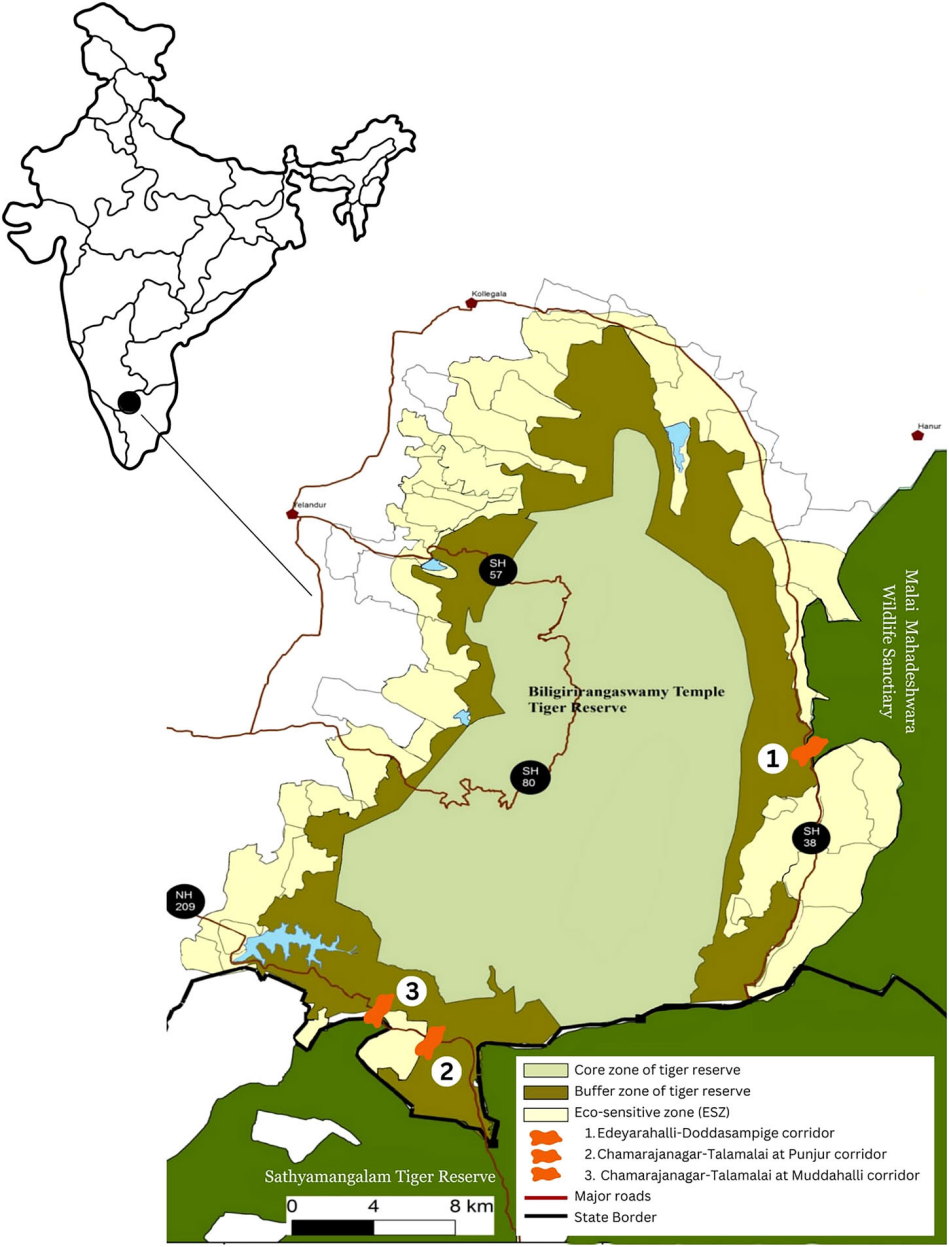
Map of Biligiri Rangaswamy Temple Tiger Reserve with the core, buffer, ESZ and location of the elephant corridors. This map is based on the ESZ notification published by the MoEFCC of the reserve published in 2019

BRT is located in the Western Ghats in South India with an area of 575 km^2^ (Fig. 1). The Malai Mahadeshwara wildlife sanctuary is located to its east while the Sathyamangalam Tiger Reserve in the neighbouring state of Tamil Nadu borders the south. Towards the north and west are agricultural lands and towns. Among the wildlife found within the PA are tigers, elephants and sloth bears. Roughly 20,000 Soligas^6^ live within and around the PA (Rai et al. 2021). In 1974, when BRT was declared a wildlife sanctuary, the Soligas were sedentarised and resettled to villages on the periphery of the PA, including into an area that has been identified as an elephant corridor. Roughly 10,000 people live in 100 villages surrounding the sanctuary (BRT Tiger Management Plan p. 15, 67).

The agrarian landscape around BRT is dominated by small and marginal rainfed farms that grow a combination of cereals, millets and vegetables. The introduction of ginger, vetiver, chia seeds, maize and other crops and their associated capital along with a burgeoning real estate market has led to changes in agricultural practices and livelihoods, which will be detailed below.

## The Multiple Pressures on Farmers

This research identifies five key overlapping factors and forces—three related to conservation and two to agrarian dynamics—that collectively contribute to the formation of the squeeze. They are 1) restrictions on access to PAs, 2) restrictions on land use outside PAs, 3) uncertain land tenure and changes in legislation, 4) insufficient or no compensation in the case of crop depredation, and 5) changing cropping patterns and volatile markets. Together, they squeeze farmers and enable the acquisition of land for elephant corridors.

### Restrictions on Access to PAs

Since BRT was designated as a wildlife sanctuary in 1974 and later declared a tiger reserve in 2011, the forest department has steadily increased restrictions on neighbouring communities’ access to resources within the PA. The collection of uncultivated greens, tubers, gooseberry, and other produce for domestic consumption and to market locally has been restricted. Grazing of livestock and gathering firewood has also been banned or heavily regulated, posing a challenge for those who rely on the forests. To reduce dependence on firewood, the forest department encouraged the switch to liquefied petroleum gas (LPG), increasing both market reliance and household expenses.

Grazing restrictions in BRT have limited pasture for native cattle, with most of the surrounding land under cultivation, and fodder costs being prohibitive. While the forest department views grazing as a threat, it recognises the gradual decline in cattle populations due to both a change in agrarian dynamics and conservation-related restrictions. A forest department official^7^, reflecting on its conservation benefits, said:

Half the population of cattle will decrease in the next ten years. None of the new generation is interested to graze cattle and go into the forest. After this generation [dies], the cattle population will come down. None of the new generation also wants to work in agriculture or do coolie work. They are going to Mysore and Bangalore. Who will be left here to do agriculture?

This reflects the forest department’s recognition that shifts within the agrarian landscape could ultimately support conservation efforts.

### Restrictions on Land Use Outside PAs

The delineation of land beyond BRT as a buffer area for the tiger reserve and an ESZ extends the influence of conservation beyond its borders. According to the WLPA, a tiger reserve must be divided into core and buffer areas, the former kept inviolate for tiger conservation, while the latter ensures its integrity and promotes human-wildlife coexistence. The buffer area, a multiple land-use area including agricultural and revenue lands, involves various state departments. Its management focuses on mainstreaming wildlife conservation outside national parks and facilitating safe wildlife dispersal into restored ecological sinks (Shukla 2012).

The tiger conservation plan for BRT (2014–15 to 2023–24) acknowledges agriculture as the primary livelihood and production sector in the buffer area and advocates for “eco-agriculture” to balance food production and wildlife conservation, though specifics are lacking. It also calls for economic incentives to safeguard wildlife concerns without detailing their nature of implementation. One objective is to “mainstream wildlife concerns” in production sectors by ensuring safeguards and gaining local support through “reciprocal commitments” (p. 268) such as curbing illicit grazing, reducing firewood collection, and participation in fire protection and anti-poaching efforts,” (Compendium of Guidelines & Circulars Issued by Director (project tiger) 2004). However, it remains unclear whether mainstreaming wildlife concerns would translate into restrictions, how it would be enforced, and who would be affected.

An ESZ serves as another mechanism to impose restrictions on surrounding landscapes. In 2011, the Ministry of Environment, Forest and Climate Change of India (MoEF&CC) published guidelines to establish ESZs around wildlife sanctuaries and national parks to act as shock absorbers and transition zones with varying levels of protection from areas of high protection to areas involving lesser protection (Ministry of Environment Forests and Climate Change 2011). These zones would be of a regulatory nature rather than prohibitive unless required. In 2019, an ESZ notification for BRT was published which included activities categorized as prohibited, regulated and promoted within 0.5 km and 6 km from the boundary (Fig. 1), affecting 49 villages (Ministry of Environment Forests and Climate Change 2019). Restrictions included tree felling, digging borewells, and ambiguously worded text about agriculture and horticulture being “permitted as per the applicable laws for use of locals.” The ambiguity around applicable laws “use of locals” remains unclear, while the ban on new borewells forces reliance on rain-fed agriculture amid increasingly unpredictable weather.

A third form of conservation governance is the eco-development zone, encompassing villages within five kilometres of the PA boundary. Its objectives include restricting grazing, promoting afforestation on farmlands, and promote LPG cylinders and renewable energy to reduce forest dependence.

These three mechanisms of delineating areas as conservation spaces have prioritised restrictions over mutual benefit for wildlife and humans, redefining access, resource rights, and livelihoods in the long-term.

### Uncertain Land Tenure and Changes in Legislation

In Karnataka, most agricultural landholders have secure tenure, but uncertainty persists for certain categories of land (Azim Premji University 2014). For example, in the area surrounding BRT, land tenure insecurity affects Soligas and other marginalised communities who were granted land by the state beginning in the late 1950s^8^. These forested lands, now known locally as *saguvalli patta*, were released for settlement. The uncertainty arises from conditional title deeds, with many owners lacking the essential Record of Tenancy Rights and Cultivation (RTC) or receiving a shared RTC for multiple families. Without such a document, selling this land is virtually impossible.

Owning *saguvalli patta* land presents several challenges: it can only be inherited, must be used for agriculture, and cannot be sold to people from outside the panchayat (pers. comm. panchayat and forest department officials). Most farmers seeking additional land within the panchayat opt to lease it, as it is a more cost-effective alternative to the high expense of purchasing land. Second, farmers without an RTC are ineligible to secure bank loans. Third, ESZ rules combined with conditional land tenure prevent farmers from digging new borewells for irrigation. Finally, farmers who face crop depredation are ineligible to apply for compensation in the absence of RTCs; more on this in the next section.

The consequences of these rules, both written and unwritten, have significantly impacted farmers. For instance, these farmers who are unable to sell land even if they wish to, cannot use land sales to repay outstanding loans, unlike revenue land holders who have benefitted from recent amendment to the Karnataka Land Reforms Act^9^. Without access to bank loans, farmers rely on high-interest informal credit, often leading to crippling debt^10^. Additionally, they must seek the forest department’s approval to dig borewells, a requirement not applicable to revenue landowners. Interviewees highlighted their applications facing long delays outright rejection, giving the forest department significant influence over agricultural practices and indirectly forcing irrigation-dependent farmers to abandon farming or relocate.

### Compensation for Crop Depredation

To protect their crops from elephants and wild pigs, farmers near BRT’s boundary often stay in their fields overnight around the harvest period. While some have installed makeshift solar fences, those without much capital have to stand guard themselves. Poorly maintained or absent elephant-proof trenches and solar fences allow easy access to farms.

To counter negative attitudes arising from crop depredation, the Karnataka Forest Department (KFD) is supposed to provide monetary compensation to farmers for crop losses from all wild animals. However, this system has several flaws. First, only damage by elephants is eligible, even though wild pigs cause far more destruction. The KFD states that farmers can claim compensation for crop damage by wild animals under the WLPA, yet only losses from elephants (a schedule I species) are compensated, excluding wild pigs (a schedule III species) despite both qualifying. Forest department officials who were asked about this said they were following orders passed down by their superiors.

Another issue is that the compensation process is complex, time-consuming, and corrupt, with farmers often needing to bribe officials for loss assessment. Those without RTCs are ineligible, and even approved claims face delays in receiving compensation.

Lastly, the compensation provided is significantly lower than the actual value of the crop lost. A 2016 government circular by the MoEF&CC recommends that compensation should be 60% of the estimated crop damage, the justification being that “If the compensation is close to 100% of the crop value there will be no incentive for the farmer to protect his crops” (Ministry of Environment Forests and Climate Change (2017) p. 17). As a result, many farmers refrain from applying, finding the payout insufficient.

These four issues underscore the challenges farmers face in protecting their crops and applying for compensation. The resulting economic hardship compounds existing agricultural difficulties, which will be discussed in the next section. Repeated crop depredation often forces farmers to abandon cultivation and seek alternative sources of income.

### Cropping Patterns and Volatile Markets

Cropping patterns around the corridors have witnessed significant changes in recent decades. Previously reliant on rainfed farming, crops cultivated included millets, pulses, traditional varieties of maize and some vegetables. In the 1970s, hybrid maize provided to Tibetan refugees by the Mysore Resettlement and Development Agency as an income generating crop were adopted by farmers due to its ability to grow under rainfed conditions and on relatively poor soils. Today, maize dominates both rainfed and irrigated farming, and has become part of the contemporary food regime, integrated into the industrial grain–oilseed–livestock complex (Jakobsen 2020), with much of the yield being converted into poultry and cattle feed. However, low profit margins, unpredictable rainfall, fluctuating market prices and wildlife depredation pose ongoing challenges.

The introduction of borewells enabled a shift from single-season to year-round farming, crop diversification, and increased fertiliser and pesticide use, while also altering tenurial regimes. Ginger, for example, was introduced to the western side of BRT in the early 2000s by farmers from Kerala who started leasing land in Karnataka. This movement of capitalist ginger farmers was a result of multiple crises in agriculture in the district of Wayanad and other parts of Kerala in the early 1990s (Münster 2015).

Ginger cultivation predominantly operates on leased land with rents varying by irrigation source and soil quality. The focus on maximising yield leads to heavy fertilisers and pesticide use, significantly impacting the region’s ecology (Punarchith 2014). Considered a high-risk crop, ginger farming is often described as a ‘gamble’ (Münster 2015) and akin to participating in the lottery as mentioned by many interviewees with price crashes and disease outbreaks causing major losses. As one farmer^11^ put it, “I got the timing of my harvest wrong so I burned my hands and my land,” referring to financial losses and soil degradation from excessive chemical use.

The hope of winning the agricultural lottery has driven many farmers in Karnataka to cultivate ginger on their own or leased land, often resulting in heavy losses and mounting debt. This gamble extends to other crops as well, with farmers relying on high-interest informal loans, sometimes reaching up to 10% per month. Unpredictable rainfall, wildlife depredation, and minimal state support further exacerbate their vulnerability. For many, daily wage labour is not a choice but a necessity to cover household and farming expenses, and for debt repayments. Those trapped in a vicious cycle often end up selling their land—revenue landholders typically to real estate speculators or those seeking to build farmhouses, while those with *saguvalli patta* land may sell to conservation actors, as seen in this case.

Many among the younger generation are leaving farming, with some taking up agricultural labour or produce transport, while other migrate to cities or towns to work in construction, garment industries, or in the gig economy. This has resulted in the “geriatrification” and “greying” of agriculture (Rigg et al. 2012; Mohanty and Lenka 2023), as the younger generation opts out due to declining farming incomes, limited access to immediate cash, hardship, and aspirations for a better life. Parents prioritise education to secure their children’s futures, as one farmer^12^ put it, “We do not want our children to struggle the way we have. We are doing all we can to educate them so they have a better future.” Similarly, a 22-year-old^13^ who had dropped out of school said:

there is no security in farming and no daily income. At least by doing coolie work or working in the garment sector there is a steady income. Many of my friends are working as food delivery riders in Bangalore and Mysore. They are making good money and are able to send some back for their parents.

As discussed, these five factors and forces exert various pressures on farmers, often pushing them to sell their land. The next section will explore how agricultural land is being acquired for elephant corridors, enabled by this squeeze.

## The Elephant Corridors

### The Muddahalli Corridor

**Fig. 2:**
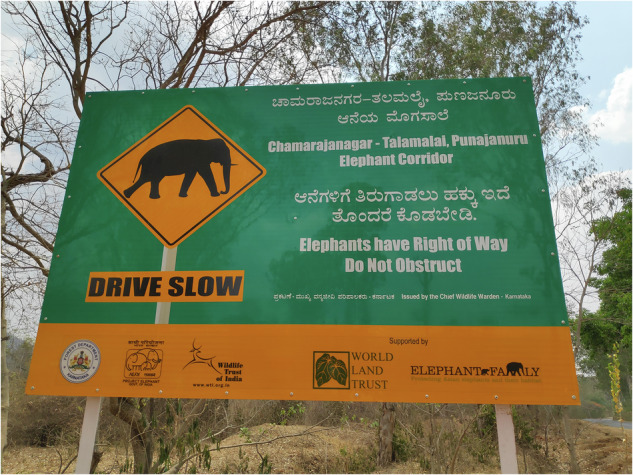
Boards such as these are erected in identified corridors

One of the three elephant corridors around BRT identified by the Wildlife Trust of India (WTI) (Menon 2005; Menon 2017) is the Chamarajanagar – Talamalai corridor at Muddahalli that connects it to the Sathyamangalam tiger reserve in Tamil Nadu via a strip of land that is 1.5 km long and 0.5 km wide. To the north and the south of the corridor are the villages of Dodda Muddahalli and Goremadu Doddi respectively. Dodda Muddahalli is predominantly inhabited by Lambani and Madiga scheduled caste communities while Goremadu Doddi is populated by Soligas. An interstate highway runs through the corridor, primarily used by vehicles transporting agricultural produce to markets in Tamil Nadu. Signboards along this highway have been put in place which let people know ‘Elephants have right of way’ (Fig. 2).

The intention to acquire land for this corridor was proposed in 2005 (Menon 2005), this despite it being *saguvalli patta* land. Support from the forest department ensured that there were no obstacles regarding the transfer of ownership. The acquisition process began in 2017 when WTI submitted a concept note^14^ to the Karnataka Forest Department, highlighting the need to secure it to prevent the loss of connectivity for tigers and elephants. The note highlights specific agricultural lands in the two villages, particularly those owned by certain families, that obstructs animal movement. To address this, the acquisition of 31.74 acres was proposed, 10 acres of which belonged to the Muddahalli Joint Farming Cooperative Society to the north of the corridor, and 21.74 acres of s*aguvalli patta* land belonging to 6 Soliga and Lambani families along the southern boundary (Table 1).

**Table 1:** Details of the land acquired for the Chamarajanagar – Talamalai at Muddahalli corridor

Owner and community/caste	Land tenure	Extent of land identified (in acres)	Extent of land sold (in acres)	Priority phase
Farmer 1 (Soliga)	*Saguvalli patta*	3.25	3.25	Phase 1
Farmer 2 (Soliga)	*Saguvalli patta*	3.25	3.25	
Farmer 3 (Lambani)	*Saguvalli patta*	3.12	3.12	
Farmer 4 (Dalit)	*Saguvalli patta*	3.03	3.03	
Farmer 5 (Lambani)	*Saguvalli patta*	5.7	4	
Farmer 6 (Lingayat)	*Saguvalli patta*	6.64	0	
Muddahalli Joint Farming Cooperative Society	Forest leased land	10	0 (at the time of fieldwork)	Phase 2

The acquisition process commenced with support from the forest department. In correspondence accessed^15^, the Principle Chief Conservator of Forests of Karnataka instructed the Deputy Conservator of Forests and Director of BRT saying “…the Wildlife Trust of India may be provided with all the support for securing the corridor in favour of the Department.” The concept note proposed a two-phase acquisition process. Phase one, deemed critical for corridor’s success, involved acquiring land or offering a land-for-land deal, though farmers claimed only monetary compensation was provided. Phase two, involving 10 acres was considered “desirable but not mandatory”. Once secured, the corridor was expected to ensure unhindered connectivity between the two tiger reserves.

The four-year land acquisition plan started with trust building efforts through dialogue and what the note calls “entry point activities”. Beginning in 2017, these efforts included a health camp as part of WTI’s Mudahalli Elephant Link project’s community support activities^16^. Additional social engagement programs to earn the community’s trust included renovating a childcare centre, distributing fuel-efficient stoves and solar lanterns to reduce depending on firewood, and holding sensitisation sessions for schools, local communities, and the forest department. Pasture development was planned; however, no progress had been made at the time of data collection. Once trust was established, discussions and negotiations on land acquisition began.

Of the six families whose lands were identified for acquisition, five were interviewed while the sixth could not be contacted. One, from the locally dominant Lambani community, sold only part of his land, unwilling to part with it entirely. Two now rely entirely on daily wage labour, while another combines daily wages with work as a land leasing agent. A fifth, with a government job is not financially dependent on the land. Despite varying financial situations, all the families expressed regret over selling their land. One farmer said^17^:

Selling the land helped us clear our debt, buy a few additional items for our house and help with our children’s education. But the compensation we got had to be split among five siblings so we each got very little. We could not even think of buying other land with that amount of money because it is so expensive. By owning the land, at least we could grow some of our food. Now we depend on the market for everything.

Other landowners shared similar stories of remorse for whom their land was more than just an asset.

Although the process of acquisition was labelled as voluntary, interviews with the families suggest otherwise^18^. The two Soliga families were told by a WTI representative that their land was actually forest land and needed to be *returned* to avoid further wildlife depredation and doing so would benefit them and neighbouring farmers. While the entry point activities aimed to manufacture consent, this messaging fostered a sense that the land was never rightfully theirs. A farmer^19^ said indignantly

First they said they that the forest [referring to BRT] was for the tiger. Now they are saying this land is for the elephant. There has been no change after they bought the land. Elephants are still destroying crops.

Land acquisition for the corridor has sparked fear and anger among neighbouring farmers. A farmer^20^ noted the increasing power of the forest department and WTI, fearing more land takeovers for conservation. “In the future, more and more land can be bought. Where will we farmers go?” he questioned. Another, whose family received a government grant in the 1950s, has been worried about losing his land due to conditional title deeds. He said, “We were told that if we stopped cultivating our land, it would be taken away. This is why we make sure to plough it every year even though we do not grow any crops so that it gives the forest department the feeling that something is being grown.” The lack of tenurial security is a source of fear stemming from the land earlier being under the possession of the forest department, a concern not shared by those with clear title deeds.

### The Edeyarahalli Corridor

**Fig. 3:**
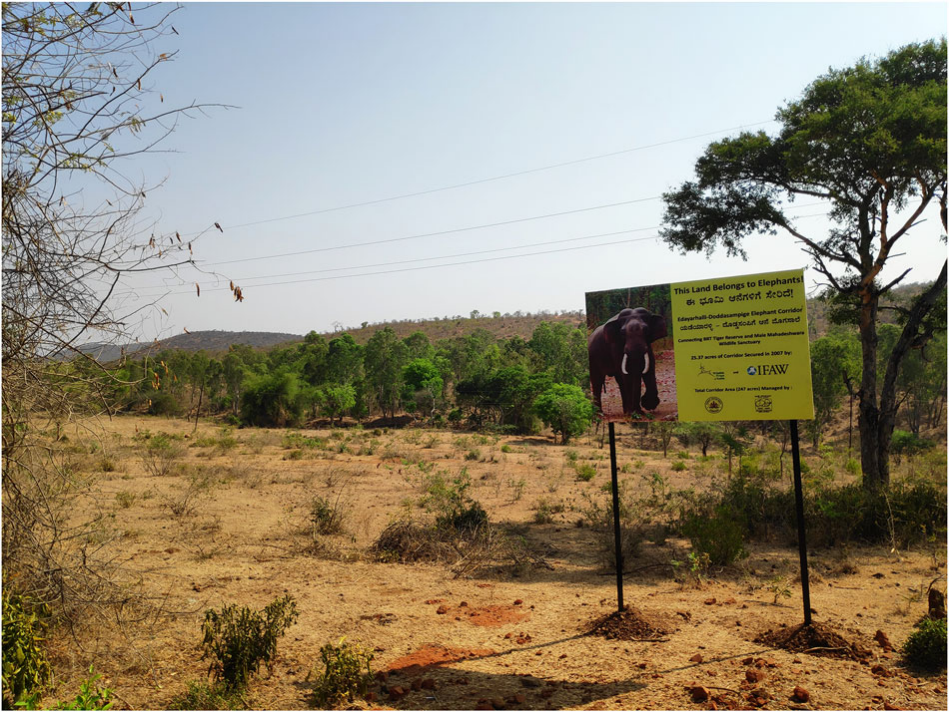
Land acquired by WTI that was subsequently transferred to the forest department

The second corridor, the Edeyarahalli – Doddasampige corridor, links the BRT Tiger Reserve to the Malai Madeshwara Wildlife Sanctuary in Karnataka via a strip of land measuring 0.5 km in length and 2 km in width (Menon 2017). In 2003, with support comparable to that received for the Muddahalli corridor, the Wildlife Trust of India (WTI) purchased 25.5 acres of *saguvalli patta* land from 17 farmers to expand the corridor. This land was later transferred to the Karnataka Forest Department in 2009 through a Memorandum of Understanding^21^. A board on the land acquired declares ‘This land belongs to elephants!’ (Fig. 3). As noted, I could not locate or speak to the original land owners.

North of this corridor, roughly 400 acres were released by the forest department in the 1980s for agriculture and settlements. While most landowners received title deeds, some with land adjoining the corridor have not been issued RTCs. A forest official^22^ interviewed confirmed plans to acquire at least 50 additional acres for the corridor. Notably, the same official acknowledged agrarian shifts and youth moving away from farming, suggesting awareness of changes that could facilitate further expansion.

### The Punjur Corridor

The Chamarajanagar – Talamalai corridor at Punjur is the third, linking the BRT Tiger Reserve in Karnataka to the Sathyamangalam Tiger Reserve in Tamil Nadu via a strip of land 3.6–4.05 km long and 0.04–0.1 km wide (Menon 2017). First identified in the mid-1980s (Sukumar 1985), the land remined forested until 1990–91, when it became a resettlement site for 250 Soliga households relocated by the forest department from 6 villages within BRT. Each household was reportedly allotted two acres of land for cultivation, though interviewees noted smaller allocations. The three resulting settlements – Srinivasapura colony, Muneeshwara colony and Hosapodu – are mapped as village enclosures within the PA and are said to “severely hindered the movement of elephants and other wild animals,” (Menon 2017, p. 620). Although it has been labelled as a “lost corridor” (KETF 2012), WTI maintains that securing 126 acres from 86 families is a priority, with additional land from Muneeshwara Colony to follow after community consultations (2017 p. 621).

In 2019, some residents of Muneeshwara colony were asked to relocate by the forest department near Nanjangud, roughly 60 km away with promises of land and access to jobs in nearby factories. However, they resisted, saying that had already been displaced once, would be far away from their gods in BRT, and raised concerns about their health and dependence on markets. Despite their reluctance, a forest department official^23^ stated “The Punjur corridor is not that easy. A lot of settlements are there. Once this corridor [the Chamarajanagar – Talamalai at Muddahalli] is done we will take up that one.” The 2023 MoEF&CC report *Elephant Corridors of India 2023* also supports securing land for this corridor.

The corridors described above are three of 101 or more that are slated to be secured in India. While land acquisition is one approach, other interventions such as zonation exercises and the imposition of rules suggest further instances of the Conservation-Agrarian Squeeze.

## Discussion and Conclusion

The critical conservation literature underscores how the area-based fortress conservation model has displaced communities, forcibly excluding those dependent on these environments for their livelihoods to prioritize wildlife preservation (Brockington 2002). This approach used in PAs is part of a powerful narrative that has used discursive technologies—recurring narratives, imagery, and practices—that legitimize certain knowledge, actors, and actions while marginalizing others (Marijnen and Verweijen 2016). These established dominant narratives seem indisputable, shaping interventions as though they reflect absolute reality (West 2006). A ‘light’ version of these discourses is slowly extending beyond PAs, as landscape-level conservation and connectivity have gained prominence, leading to a conservation-driven governance approach that imposes increasing restrictions beyond PA boundaries. Here, incremental changes introduced, including the gradual loss of access to resources make land ‘grabbable’ (Benjaminsen and Bryceson 2012; Weldemichel 2022) with hegemonic processes preceding eventual dispossession. While these lands may not be taken over directly for conservation in the short term, they are increasingly governed by its logic.

It is here that this paper contributes to the existing literature by exploring how, even beyond PAs, discursive technologies—specifically land acquisition for elephant corridors—along with conservation restrictions and gradual agrarian shifts, collectively drive dispossession and marginalize certain actors. Around BRT and other Indian landscapes, crisis narratives emphasizing the need for connectivity for megafauna are expanding conservation spaces through corridors. In this case, landowners were convinced that their land needed to be *returned* to the forest department and doing so would help mitigate human-wildlife conflict, although this has made no difference in the case of the latter. While the existing literature referenced in the introduction explores various processes and actors driving a squeeze, this article specifically examines how conservation interventions and agrarian dynamics converge to reshape livelihoods and support systems.

The acquisition of land for elephant corridors exemplifies the expansion of conservation spaces beyond protected areas and into neighbouring agrarian landscapes. Using the concept of the CAS, I have highlighted five factors and forces that drive the enclosure of land identified for connectivity, facilitated by distress in agrarian landscapes. These are the restrictions on access to PAs, restrictions on land use outside PAs, uncertain land tenure and changes in legislation, compensation for crop depredation, and cropping patterns and volatile markets. Although farmers experience each of these challenges individually, they are interconnected and centre around access, land tenure, and finance. The intersection of conservation efforts and agrarian dynamics also raises critical questions about land use, history and power. The factors and forces listed here, while not exhaustive, are those that emerged from the empirical material and can be highly contextual and historical. In addition, while those impacted in this case were primarily tribals, people from varied backgrounds could also be affected. An interesting aspect of this case is also the nexus between state and non-state actors, facilitated by these five ‘technologies of rule’, which creates conditions for consolidating ‘territorialization for conservation’, as highlighted by Kabra and Das (2022).

Conceptually, the CAS draws on four related terms – the grab, induced volition, rendering surplus, and expulsion – while also differentiating itself from them in the following ways. While comparable to land and green grabs, and induced volition, the CAS is distinguished by the fact that land enclosure is driven by a combination of factors in conservation and agrarian landscapes that facilitate the grab. Similarly, rendering populations as surplus has been used with specific reference to largescale agricultural expansion and capital accumulation that leads to dispossession. The CAS, however, highlights a variety of factors and forces while focusing on smaller-scale land deals. This does not imply that the CAS is inapplicable to larger-scale land deals. Finally, the CAS is most similar to the concept of expulsion in terms of knowledge forms, complexities, and the convergence of economic forces that lead to it. What is missing is the conservation logic that governs these landscapes, both within and along the periphery of such PAs.

The CAS should be viewed within the larger framework of the increasing conservation emphasis on improving connectivity between PAs and extending conservation frontiers through the approaches discussed in the introduction. With a specific type of territoriality for conservation gaining in popularity, the CAS has the potential to travel to other contexts in the global south, particularly in areas with a farm-forest frontier. Calls to set aside more land for biodiversity conservation would likely put increasing pressure on agrarian livelihoods resulting in more examples of the squeeze, while discounting questions of livelihoods, food security, and food sovereignty. With precise figures on those displaced from within PAs itself unreliable (Lasgorceix and Kothari 2009), quantifying those impacted by the squeeze would be next to impossible. The lack of evidence about the negative social impacts of conservation is no longer the challenge. The real challenge lies in reimagining conservation to be more just, equitable and inclusive.

## Data Availability

No datasets were generated or analysed during the current study.

## Footnotes

1 https://wiienvis.nic.in/Database/Protected_Area_854.aspx (accessed on 04/03/2024)

2 Karnataka Private Conservancies Rules 2018

3 I use inverted commas for “secure” to underscore the unequal power dynamics and coercive tools involved in land acquisition and dispossession.

4 Edeyarahalli – Doddasampige, Chamrajanagar – Talamalai at Muddahalli, and Chamrajanagar – Talamalai at Punjur corridors in Menon et al. 2017

5 Village council

6 A Scheduled Tribe

7 interviewed on 21/01/2022

8 An example is 600 acres granted to Harijans (now Dalits) and Lambanis by the agriculture and forest departments of the former Mysore state.

9 The 2020 amendment allows non-agriculturists to acquire agricultural land, a right previously restricted.

10 Extensive data on informal debt sources was unavailable during the study.

11 Interviewed on 29/04/2022

12 Interviewed on 10/05/2022

13 Interviewed on 10/05/2022

14 Wildlife Trust of India (2017). Accessed at the office of the Deputy Conservator of Forests in Chamarajanagar

15 Accessed at the office of the Deputy Conservator of Forests in Chamarajanagar

16 https://www.wti.org.in/news/mudahalli-elephant-link-health-camp-organised-for-corridor-dependent-villages/ (accessed on 04/10/2021)

17 Interviewed on 14/05/2022

18 For another corridor where land was acquired, WTI note that they “persuaded 30 people to voluntarily relocate”. Bathija and Sylvander (2023) argue that framing dispossession as voluntary legitimises it as an acceptable conservation strategy.

19 Interviewed on 09/05/2022

20 Interviewed on 10/05/2022

21 Though the acquisition preceded Menon et al. (2005), the process of identifying and securing corridors began in 2001, first mentioned by Sukumar (1985).

22 interviewed on 21/01/2022

23 Interviewed on 26/04/2022
